# Triploid Citrus Genotypes Have a Better Tolerance to Natural Chilling Conditions of Photosynthetic Capacities and Specific Leaf Volatile Organic Compounds

**DOI:** 10.3389/fpls.2020.00330

**Published:** 2020-04-21

**Authors:** Radia Lourkisti, Yann Froelicher, Stéphane Herbette, Raphael Morillon, Félix Tomi, Marc Gibernau, Jean Giannettini, Liliane Berti, Jérémie Santini

**Affiliations:** ^1^CNRS, Equipe de Biochimie et Biologie Moléculaire du Végétal, UMR 6134 SPE, Université de Corse, Corsica, France; ^2^CIRAD UMR AGAP, Station INRA, Corsica, France; ^3^UCA, INRA, PIAF, Clermont-Ferrand, France; ^4^Equipe “Amélioration des Plantes à Multiplication Végétative”, UMR AGAP, Département BIOS, CIRAD, Petit-Bourg, Guadeloupe; ^5^CNRS, Equipe Chimie et Biomasse, UMR 6134 SPE, Université de Corse, Corsica, France

**Keywords:** polyploidy, photosynthesis, chlorophyll fluorescence, oxidative metabolism, volatile organic compounds, cold stress

## Abstract

Low temperatures during winter are one of the main constraints for citrus crop. Polyploid rootstocks can be used for improving tolerance to abiotic stresses, such as cold stress. Because the produced fruit are seedless, using triploid scions is one of the most promising approaches to satisfy consumer expectations. In this study, we evaluated how the triploidy of new citrus varieties influences their sensitivity to natural chilling temperatures. We compared their behavior to that of diploid citrus, their parents (Fortune mandarin and Ellendale tangor), and one diploid clementine tree, as reference, focusing on photosynthesis parameters, oxidative metabolism, and volatile organic compounds (VOC) in leaves. Triploid varieties appeared to be more tolerant than diploid ones to natural low temperatures, as evidenced by better photosynthetic properties (*P_net_, g_s_, F_v_/F_m_*, ETR/*P*_net_ ratio), without relying on a better antioxidant system. The VOC levels were not influenced by chilling temperatures; however, they were affected by the ploidy level and atypical chemotypes were found in triploid varieties, with the highest proportions of *E*-β-ocimene and linalool. Such compounds may contribute to better stress adaptation.

## Introduction

Citrus is one of the main fruit crops in the world and it has nutritional, economic, and medicinal importance. The Mediterranean region is the largest exporter of fresh market citrus fruits (FAO, 2017). This market is highly competitive, with a large volume of clementines and oranges produced in Spain, Morocco, or Italy, with a marked seasonality. In order to develop market opportunities, citrus breeding programs have focused mainly on innovative small citrus which produce seedless fruit with a different maturity period and interesting pomological, agronomical, and organoleptic traits. Alongside these new consumer preferences, climate changes have triggered the development of new varieties and highlighted the need to assess plant adaptation to abiotic constraints. Abiotic stresses, like drought or extremes temperatures, adversely affect the quality of citrus production, yield, and growth. In this context, the selection of new citrus varieties, with traits that improve adaptation to abiotic stresses, can contribute significantly to the development of the citrus fresh fruit market. Among Mediterranean countries, France stands out for its clementine production, with 97% produced in Corsica (30,000 tons per year). The Corsican production is characterized by endemic products with a great organoleptic quality thanks to the island producers and climate, resulting in it being given protected geographical indication (PGI). The Corsican clementine is mainly produced from October to January while Corsican pomelo production begins from April to June. Thus, it would be beneficial to boost the citrus fresh fruit market by extending the production period with small innovative seedless citrus from January to April.

Triploidy could play an important role in the coming decades by improving fruit traits, biomass, and abiotic stress tolerance resulting in commercial benefits (Esen and Soost, 1971; Aleza et al., 2010; Costa et al., 2019). Most citrus species are diploid, with a basic chromosome number *x* = 9 (2x = 18) (Krug, 1943). However, spontaneous autotetraploid have been found among citrus cultivars, while a single triploid formation has occurred in ‘Tahiti’ lime (Lee, 1988). Triploidy in citrus can occur by sexual hybridization between diploid parents (Esen and Soost, 1971) or between diploid and tetraploid parents (Esen et al., 1978). Triploid hybrids arising from diploid × diploid hybridizations are produced through the formation of unreduced gametes, usually by the female parent, with a relatively low frequency which varies among different genotypes. For example, Navarro et al. (2015) showed that the ‘Fortune’ mandarin – when used as a female parent – produced a greater frequency of triploid hybrids per fruit than clementines. In citrus cultivars, triploid hybrids have been successfully used to produce seedless fruits (Aleza et al., 2012; Rouiss et al., 2018). Despite their desirable characteristics, such as larger fruit and higher yield (Hoshino et al., 2011), triploid plants are unusual because of their inviable seeds. Triploid citrus are heterozygous and each hybrid is unique in specific allelic combination. Thus, phenotypic traits of triploid hybrids compared to diploid hybrids may be conferred by the expression of specific alleles from parents or by ploidy. This can lead to high gene dosage, gene expression pattern ranging from additive expression from each allele to expression dominance by a single allele, to biases toward a specific parental genome (Madlung, 2013). Polyploidy in citrus is associated with an increase in stomata size and decrease in density (Allario et al., 2011). Although maximal gas exchange had been related to low stomatal size and high density (Franks and Beerling, 2009), some authors concluded that polyploidy *per se* may not necessarily have a reduced gas exchange capacity and can also increase adaptive response to stressful environmental conditions (Ramsey, 2011; Madlung, 2013). Moreover, it has been suggested that hybridization results in great genomic changes that contribute to better and faster adaptation to a novel environment (Wang et al., 2006; Chen, 2007). To our knowledge, no study has been conducted on triploid tolerance to adverse environmental conditions. Yet, the use of tetraploidy, and more generally polyploidy, appears to be a relevant alternative pathway for developing plants that are more tolerant to biotic and abiotic stresses, without impacting fruit yield and quality (Aleza et al., 2012). Several studies revealed that citrus tetraploid seedlings, or used when grafted, were more tolerant to abiotic stresses (salt stress, water deficiency, nutrient disruption, and chilling stress) than diploid ones (Saleh et al., 2008; Allario et al., 2013; Ruiz et al., 2016; Vieira et al., 2016; Oustric et al., 2019).

Among abiotic stresses, chilling stress is a critical factor for citrus fruit production. Chilling stress decreases the CO_2_ assimilation and the stomatal conductance leading to disruption of photosynthesis and electron transport through the thylakoid membrane, resulting in cellular damage (Allen and Ort, 2001; Hussain et al., 2018). Indeed, the decline in photosynthesis leads to excess energy in photosystem II and I (PSII and PSI) and, consequently, to photoinhibition (Baker, 2008). The excess energy, which is not safely dissipated, can induce the overproduction of reactive oxygen species (ROS), like superoxide anion, hydroxyl radical, or hydrogen peroxide, leading to severe oxidative damage (Gill and Tuteja, 2010). Chilling stress causes a disruption in cell membrane structure that can lead to cellular electrolyte leakage (Oustric et al., 2017; Wang et al., 2017). Plants have developed a complex defense system that includes enzymes and antioxidant molecules to avoid or reduce chilling injuries. The key role of these antioxidant mechanisms that provide plants with their cold tolerance has been largely described (Santini et al., 2013; Oustric et al., 2017; Agurla et al., 2018; Hussain et al., 2018). Abiotic stresses also modify the biosynthesis and emission of volatile organic compounds (VOCs) (Loreto and Schnitzler, 2010; Peñuelas and Staudt, 2010; Vieira et al., 2016). VOCs include a wide variety of chemical compounds, with terpenes being the largest and most diverse family (Tholl, 2015). Metabolic pathways and emissions of VOCs were assumed to be temperature-dependent (Copolovici et al., 2012) but their emission significantly increases in stressed plants (Guenther et al., 2006). Some studies revealed that VOCs (sabinene, *E*-β-ocimene, linalool) are involved in biotic and abiotic stress responses including huanglongbing-associated bacterium, *Candidatus liberibacter asiaticus* (Hijaz et al., 2016), drought stress (Vieira et al., 2016), winter flooding, and salinity (Velikova et al., 2012). Some authors argued that terpenes may act as signal molecules and antioxidants (Peñuelas and Staudt, 2010; Possell and Loreto, 2013). Vickers et al. (2009) reported that terpenes can also act indirectly as membrane stabilizers reducing lipid peroxidation and, thus, the cell’s oxidative state. However, to our knowledge, no study has examined the impact of ploidy combined with temperature variations on the VOC profile.

VOCs have been widely studied in several citrus species during the chemical make-up of leaf essential oils (EO) (Lota et al., 2001; Fanciullino et al., 2006; Espina et al., 2011; Santos et al., 2015). Considering all the aspects mentioned previously, triploidy could be a promising way to both improve abiotic stress tolerance and produce seedless fruits. To take into account consumer expectations, hybridization between the Fortune mandarin and the Ellendale tangor was performed several years ago and the generated hybrids were grown in an experimental orchard on the island of Corsica. The Fortune mandarin parent was selected for its pomological traits and, used as a female, it results in a high proportion of triploid hybrids, while the Ellendale tangor parent was chosen for its organoleptic quality and its later fruit production (April).

The first aim of our study was to assess triploid varieties for the tolerance of their photosynthetic activities to chilling stress, in comparison with diploid ones obtained from the same cross. Then, we analyzed the biochemical responses of the different lines under chilling conditions in order to identify markers related to this tolerance in 3x varieties. We compared triploid and diploid hybrid citrus behavior with both parents and the diploid clementine tree. To evaluate the response of the selected varieties between a cold period and a warm one, we analyzed some properties related to their photosynthesis activity [net photosynthesis (*P*_net_), stomatal conductance (*g*_s_), electron transpiration rate (ETR), maximal quantum efficiency of PSII within dark-adapted leaves (*F_v_/F_m_*), and effective quantum efficiency of PSII within light-adapted leaves (*Φ_PSII_*)], their oxidative metabolism (hydrogen peroxide, malondialdehyde and antioxidant enzymes and compounds) and their changes in leaf VOC levels.

## Materials and Methods

### Plant Material and Growth Conditions

The experiment was carried out on 16-year-old diploid (2x) and triploid (3x) hybrid citrus trees. Scion hybrids were the result of hybridization between the Fortune mandarin (*Citrus reticulata Blanco*) and the Ellendale tangor [*Citrus reticulata Blanco x Citrus sinensis (L) Osb.].* Diploid and triploid scions were grafted onto C-35 Citrange rootstocks (*Citrus sinensis ‘Ruby Blood’ x Poncirus trifoliata).* C-35 was chosen for its tolerance to biotic (Tristeza, phytophtora) and abiotic (cold, drought) stress. C-35 seedlings used for the experiment were strictly chosen in the nursery to eliminate off-types. All trees were grown in an experimental orchard with the same South orientation and a similar height above ground. The orchard is located in San Giuliano (INRA-CIRAD), Corsica, France (42°17′05′′ N, 9°31′26′′ E) and is composed of 40 triploid hybrids and 40 diploid hybrids. In order to select genotypes with contrasting behavior within this population, these 80 genotypes were screened first using malondialdehyde (MDA) as a marker of stress tolerance in leaves sampled in September 2017 (see Supplementary Figure S1) in which the minimal and maximal mean temperatures of the month were 17.6°C and 26.6°C, respectively. Based on this screen, 2x and 3x hybrids with the highest MDA values (D1-2x, T1-3x, and T3-3x) and others with the lowest values (D39-2x, T38-3x, and T40-3x) were selected for further experiments. In addition, both parents (Fortune mandarin and Ellendale tangor) and a common clementine (*Citrus clementina* Hort. Ex Tan; SRA 92) were included in the experimental plot. Using the nine selected genotypes, physiological measurements and samplings were carried out in February 2018 (cold period) and in September 2018 (warm period) to decipher the impact of cold on trait tolerance. To that aim, we focused on the coldest and the hottest sunny days during these two periods. Meteorological data were collected throughout the sampling period (Table 1).

**TABLE 1:** Meteorological data of cold and warm periods at the experimental plot.

**Sampling period**	**Sampling day**			**Sampling month**		
		
	**Minimum temperature (°C)**	**Maximal temperature (°C)**	**Mean Temperature (°C)**	**Minimum temperature (°C)**	**Maximal temperature (°C)**	**Mean temperature (°C)**
Cold period	0.1	7.0	2.3	5.7	12.8	7.3
Warm period	21.6	30.5	25.0	19.8	28.5	24.2

### Gas Exchange and Chlorophyll Fluorescence Measurements

The main photosynthetic traits were measured on the same leaves. Twelve fully expanded mature leaves per tree were used (12 independent biological replicates). Fully expanded leaves were selected on 1-year old branches subjected to the same light exposure (South). Each parameter was measured between 9 am and 11 am.

Net photosynthesis rate (*P*_net_) and stomatal conductance (*g*_s_) were measured using an LC-PRO-SD portable infra-red gas analyzer (ADC, BioScientific Ltd., Hoddeston, United Kingdom). During the experiment, photosynthetically active radiation (PAR) was applied at the leaf surface and fixed at 1400 μmol.m^–2^.s^–1^ (Santini et al., 2013; Oustric et al., 2017; Ruiz et al., 2018). Leaf temperature was set at 25°C and ambient carbon dioxide concentration (CO_2_) was used (390 μmol. mol^–1^).

The maximum quantum efficiency of PSII (*F_v_/F_m_*), the effective quantum yield of PSII (*Φ_PSII_*), and the electron transport rate (ETR) were monitored using an OS1p chlorophyll fluorimeter (Opti-Sciences, Inc. Hudson, United States). *F_v_/F_m_* was monitored on dark-adapted leaves using clips through the thylakoid membrane for 30 min (Oustric et al., 2017). For the fluorescence measurements in the light, the fluorimeter was equipped with an open leaf-clip suitable for measurements on light-adapted leaves. *Φ_PSII_* was evaluated as described by Genty et al. (1989) and ETR (also known as J) was expressed according to Krall and Edwards (1992).

### Biochemical Assays

Four samples were collected for each genotype and for each period (four independent biological replicates). Each biochemical sample was obtained by pooling 15 fully expanded leaves selected from one individual tree for each variety and for each period. Harvested samples were immediately immersed in liquid nitrogen, ground to a fine powder, and then stored at −80°C.

Malondialdehyde content was assayed according to Hodges et al. (1999) and adapted to citrus samples as described by Santini et al. (2013). Eighty milligrams of leaf powder were homogenized in 2 mL of 80% ethanol (v/v). Homogenates were centrifuged at 3000 *×* g at 4°C for 10 min. Absorbance was determined at 440, 535, and 600 nm against a blank.

Measurements of hydrogen peroxide content were carried out using a PeroxiDetect Kit (Sigma Aldrich, St. Louis, MO, United States) according to Jiang et al. (1991). The reaction is based on the oxidization of Fe^2+^ to Fe^3+^ ions by hydrogen peroxide in aqueous solutions with an acidic pH. Leaf powder (150 mg) was homogenized in 300 μL of distilled water and centrifuged at 21,000 *×* g for 15 min at 4°C. Twenty μL of homogenates were added to each well of a 96-well microplate. A working color reagent was prepared by mixing 1 volume of 25 mM ammonium sulfate in 2.5 M sulfuric acid with 100 volumes of 125 μM xylenol orange (Sigma-Aldrich) in 100 mM sorbitol. A sample of 200 μL of the working color reagent was added to each well, and then the microplate was incubated for 1 h at room temperature. Absorbance was read at 560 nm with a microplate reader (MULTISKAN FC^TM^, Thermo Scientific, Waltham, MA, United States). The hydrogen peroxide concentration was determined from a standard curve.

Ascorbic acid content was determined as described by Stevens et al. (2008). Leaf powder (150 mg) was homogenized in 600 μL of 6% ice-cold trichloroacetic acid (w/v). Homogenates were centrifuged at 13,000 *×* g at 4°C for 15 min. Absorbance was read at 550 nm with a microplate reader (MULTISKAN FC^TM^, Thermo Scientific, Waltham, MA, United States). Total and reduced ascorbic acid content were determined using a standard curve.

Proline content was assayed according to Carillo and Gibon (2011). Forty milligrams of leaf powder were homogenized in 70% ethanol (v/v). Homogenates were centrifuged at 15,000 *× g* at 4°C for 15 min. The absorbance was read at 520 nm with a microplate reader (MULTISKAN FC^TM^, Thermo Scientific, Waltham, MA, United States). Proline content was determined using a standard curve.

For antioxidant enzymatic activities, leaf powder (54 mg) was homogenized in 2 mL of extraction buffer (0.1 M potassium phosphate, pH 7.5) and the homogenates were centrifuged at 13,000 *× g* for 30 min at 4°C. The supernatant was collected and was used for all enzymatic assays and for protein determination (Bradford, 1976). Superoxide dismutase, catalase, ascorbate peroxidase, and dehydroascorbate reductase assays were performed as described by Santini et al. (2013). Time-course measurements were monitored using a V-630 spectrophotometer (Jasco Inc., Tokyo, Japan).

### Leaf Essential Oils Extraction and Terpenes Analysis

One hundred grams of fully expanded leaves per genotype (three independent biological replicates) with the same southern exposure were harvested on each sampling day and then quickly hydrodistilled. EOs were extracted from fresh leaves by hydrodistillation and then were analyzed using gas chromatography with flame ionization detector (GC-FID) and gas chromatography/mass spectrometry (GC/MS).

Gas chromatography analysis was performed on a PerkinElmer Clarus 500 gas chromatograph (FID) equipped with two fused silica gel capillary columns (50 m × 0.22 mm, film thickness 0.25 μm), BP-1 (polydimethylsiloxane), and BP-20 (polyethylene glycol). The oven temperature was programmed to increase from 60 to 220°C at 2°C/min and then held at 220°C for 20 min. Injector temperature and detector temperature were set at 250°C with split of 1/60. Hydrogen (0.8 mL/min) was used as carrier gas. The relative proportions of the oil constituents were expressed as percentages obtained by peak area normalization, without using correcting factors. Retention indices (RIs) were determined relative to the retention times of a series of *n*-alkanes with linear interpolation (‘Target Compounds’ software from PerkinElmer).

For mass spectrometry, the EOs were analyzed with a PerkinElmer TurboMass detector (quadrupole), directly coupled to a PerkinElmer Autosystem XL, equipped with a fused silica gel capillary column (50 m × 0.22 mm i.d., film thickness 0.25 μm), BP-1 (dimethylpolysiloxane). The oven temperature was programmed to increase from 60 to 220°C at 2°C/min and then held at 220°C for 20 min. Injector temperature and detector temperature were set at 250°C with split of 1/60. Helium (0.8 mL/min) was used as carrier gas. The ion source temperature of the mass spectrometer was set at 250°C with an ionization energy of 70 eV. Electron ionization mass spectra were acquired over the mass range 40–400 Da.

### Statistical Analysis

Data were expressed as mean values ± SE and analyzed with R statistical software^1^. Two-way ANOVAs and multiple mean comparisons were carried out with the least significant difference (LSD) test at *P* < 0.05. Data were normalized and used in principal component analysis (PCA) and hierarchical clustering classification using FactomineR package (Le et al., 2008). PCA was conducted to define a relationship between physiological, biochemical, and chemical parameters and genotypes during the cold and warm period. PCA and hierarchical clustering classification helps us to better understand the similarity between variables and individuals. For chemical data, cluster analysis was conducted using Ward’s method in normalized data to obtain a hierarchical distribution of varieties and optimal number of clusters.

## Results

### Warm Period *vs*. Cold Period

**FIGURE 1:**
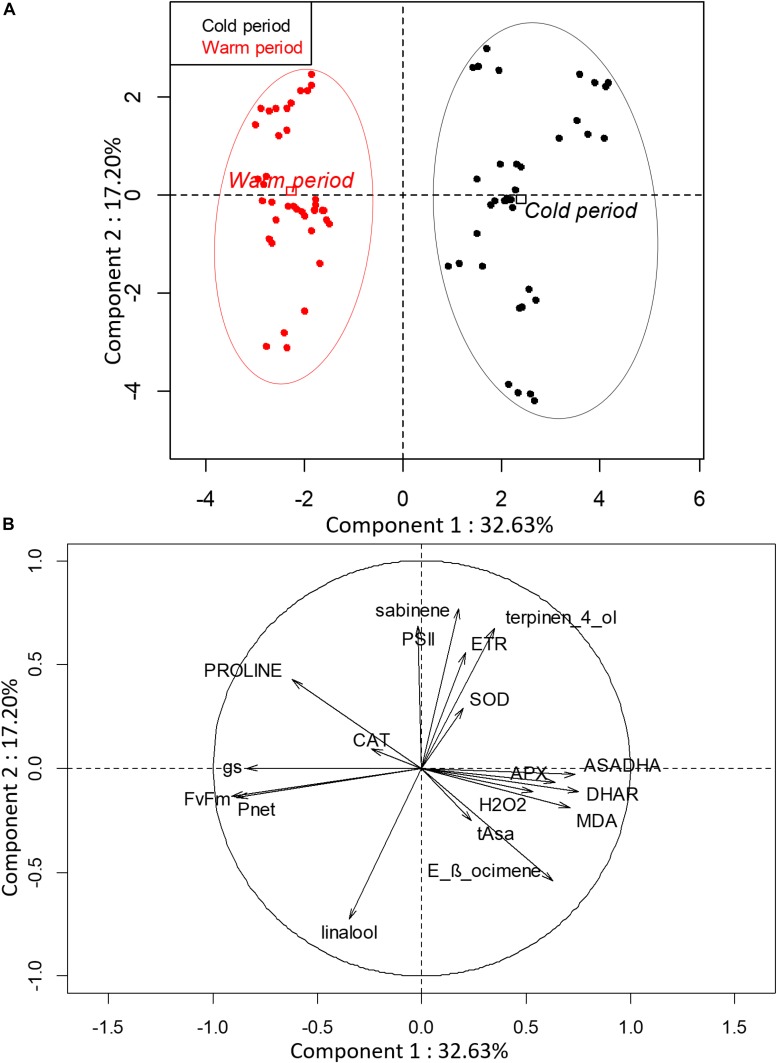
Biplot obtained from PCA performed on leaves of nine citrus varieties during cold and warm period. **(A)** Repartition of varieties on the two first axes and **(B)** contribution of investigated parameters to the two first axes of PCA. (PSII: *Φ_PSII_*).

Principal component analysis was performed with the parameters collected during cold and warm periods for the nine selected varieties to highlight the potential differences between both periods (Figure 1). The first two components explained 49.83% of the total variance of the population. Component 1 was positively correlated with oxidative metabolism parameters (Asa/DHA, DHAR, APX, MDA, and H_2_O_2_) and negatively correlated with photosynthesis parameters (*F_v_/F_m_*, *g*_s_ and *P*_net_) and proline content. Component 2 was positively correlated with *Φ_PSII_*, ETR, SOD, sabinene, and terpinen-4-ol, and negatively correlated with linalool and *E*-β-ocimene. Interestingly, the oxidative markers (H_2_O_2_ and MDA) were negatively correlated with proline content. PCA clearly splits varieties into two distinct groups according to the cold and warm period.

### Changes in Photosynthetic Parameters

**FIGURE 2:**
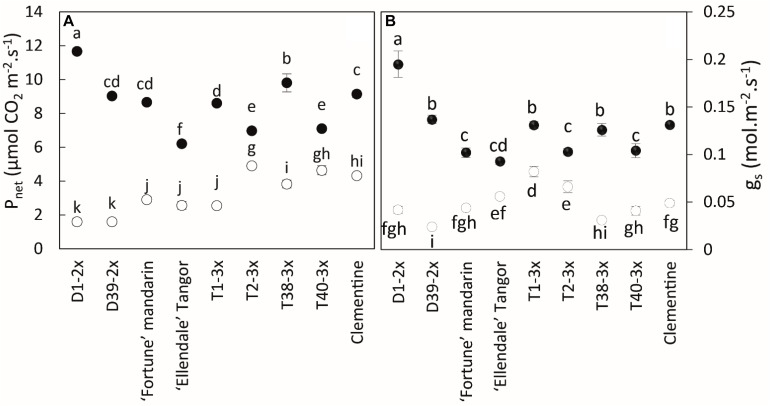
Comparison of **(A)** net photosynthetic rate (*P*_net_), **(B)** stomatal conductance (g_s_) between nine Citrus varieties during warm period (black point) and cold period (white point). All data are mean values (±SE) of 12 independent biological replicates for each genotype (*n* = 12). Data were analyzed using ANOVA and Fisher LSD tests (*P* < 0.05). Different lowercase letters indicate significant differences between the varieties and the sampling periods.

During the warm period, all varieties had very high net photosynthesis rates (*P*_net_) and stomatal conductance (*g*_s_) (Figure 2).

**FIGURE 3:**
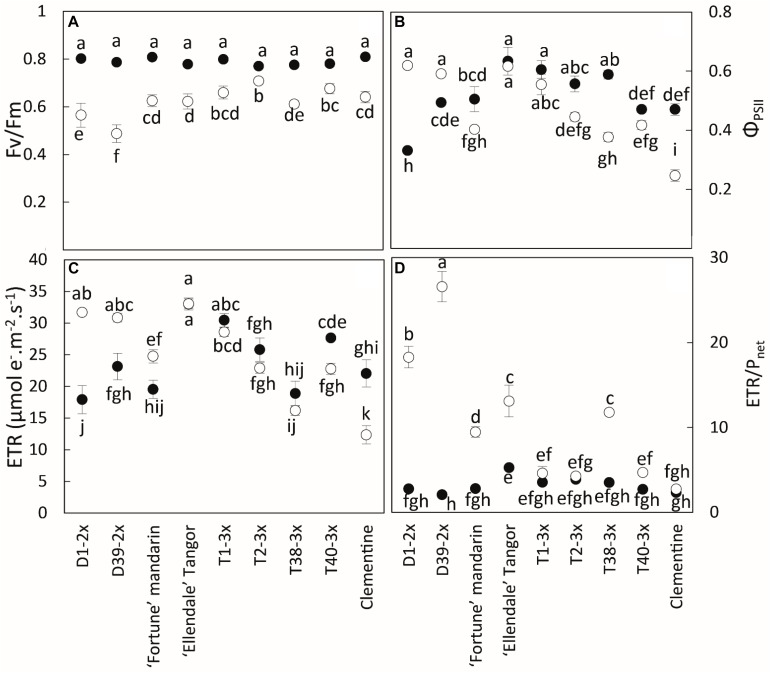
Comparison of **(A)** chlorophyll fluorescence (*F_v_/F_m_*), **(B)** quantum yield of electron transfer at PSII (*Φ_PSII_*), **(C)** electron transport rate (ETR), and **(D)** ETR/*P*_net_ ratio between nine citrus varieties during warm period (black point) and cold period (white point). All data are mean values (±SE) of 12 independent biological replicates for each genotype (*n* = 12). Data were analyzed using ANOVA and Fisher LSD tests (*P* < 0.05). Different lowercase letters indicate significant difference between the varieties and the sampling periods.

During the cold period, all varieties had lower values of *P*_net_, *g_s_*, and *F_v_/F_m_*, although the triploid varieties were less affected by chilling conditions than the diploid ones (Figures 2, 3A). T2-3x had high rates of *P*_net_ and *g*_s_ during the cold period while the lowest values were found in the diploid varieties (D2-2x, D39-2x and parents), except for clementine. The highest values of *F_v_/F_m_* (Figure 3A) were also found in T2-3x while the diploid hybrids had the lowest values. No significant difference was found between clementine, both parents, and some triploid varieties (T1-3x and T38-3x).

During the cold period, the highest values of *Φ_PSII_* were found in T1-3x and some diploid varieties (D1-2x, D39-2x) (Figure 3B). ETR was lower in some triploid varieties (T2-3x, T38-3x, and T40-3x) during the cold period (Figure 3C). The highest value was found in Ellendale tangor, no matter the period. During the cold period, clementine had the lowest value for *Φ_PSII_* and ETR.

As a whole during the cold period, triploid varieties, except T38-3x, and clementine had the lowest ETR/*P*_net_ values (∼4) (Figure 3D).

### Differences in Oxidative Metabolism

**FIGURE 4:**
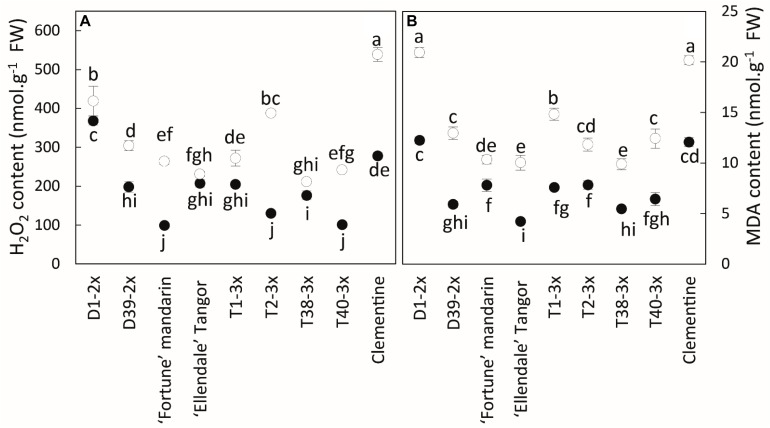
Comparison of **(A)** hydrogen peroxide (H_2_O_2_) and **(B)** malondialdehyde (MDA) contents between nine citrus varieties during warm period (black point) and cold period (white point). All data are mean values (±SE) of 4 independent biological replicates for each genotype (*n* = 4) obtained by pooling 15 fully expanded leaves. Data were analyzed using ANOVA and Fisher LSD tests (*P* < 0.05). Different lowercase letters indicate significant differences between the varieties and the sampling period.

H_2_O_2_ and MDA contents were higher during the cold period in all varieties (Figure 4). Significant differences were found between diploid and triploid varieties, but there were also contrasting values in triploid varieties. T38-3x had the lowest values for both parameters during the cold period.

**FIGURE 5:**
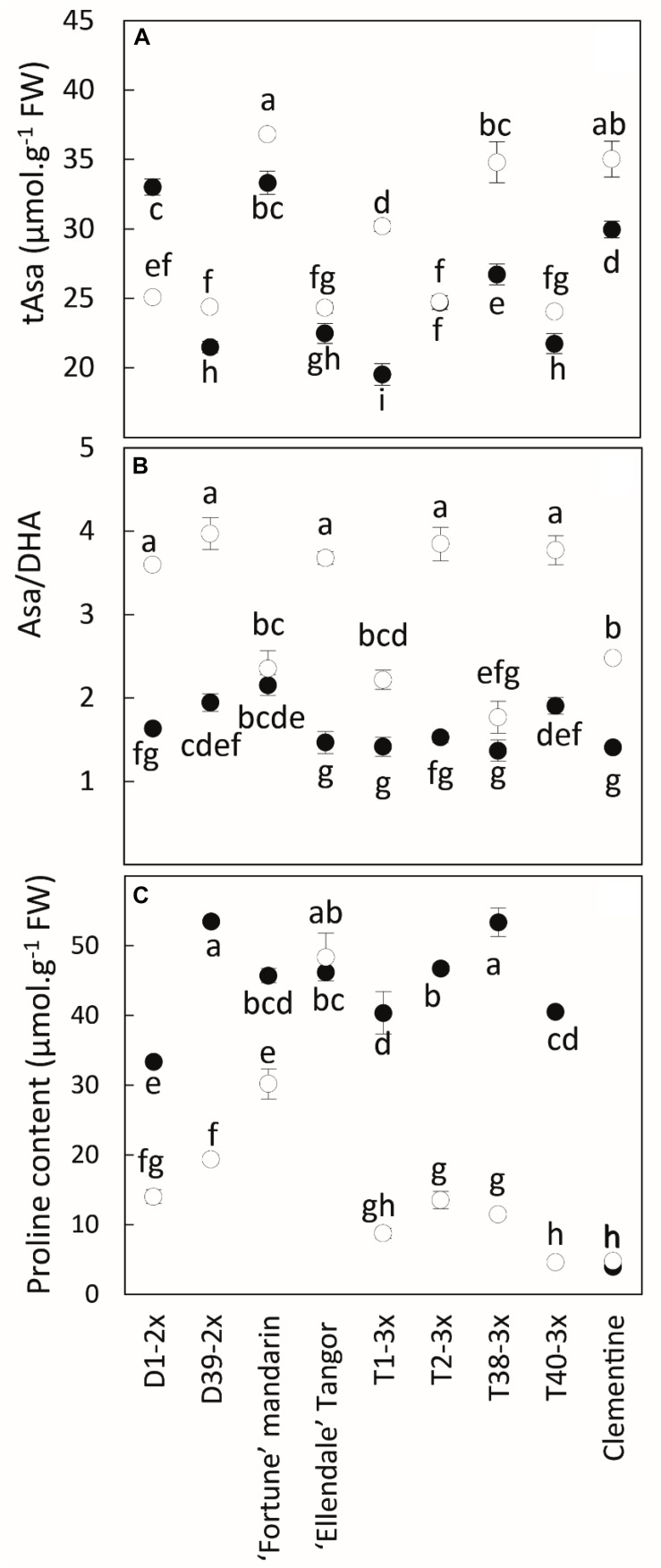
Comparison of **(A)** total ascorbate content (tAsa), **(B)** redox status (Asa/DHA) and **(C)** proline content between nine citrus varieties during warm period (black point) and cold period (white point). All data are mean values (±SE) of 4 independent biological replicates for each genotype (*n* = 4) obtained by pooling 15 fully expanded leaves. Data were analyzed using ANOVA and Fisher LSD tests. Different lowercase letters indicate significant differences between the varieties and the sampling periods.

During the cold period, total ascorbate (tAsa) was increased in all varieties except for D1-2x (Figure 5A). The content was high in Fortune mandarin, clementine, and T38-3x while no significant difference was found between the other diploid and triploid varieties. Increased values of Asa/DHA ratio were observed during the cold period (Figure 5B).

Low temperatures induced a significant decrease in proline content except for Ellendale tangor, in which no significant variation was observed between both periods (Figure 5C).

**FIGURE 6:**
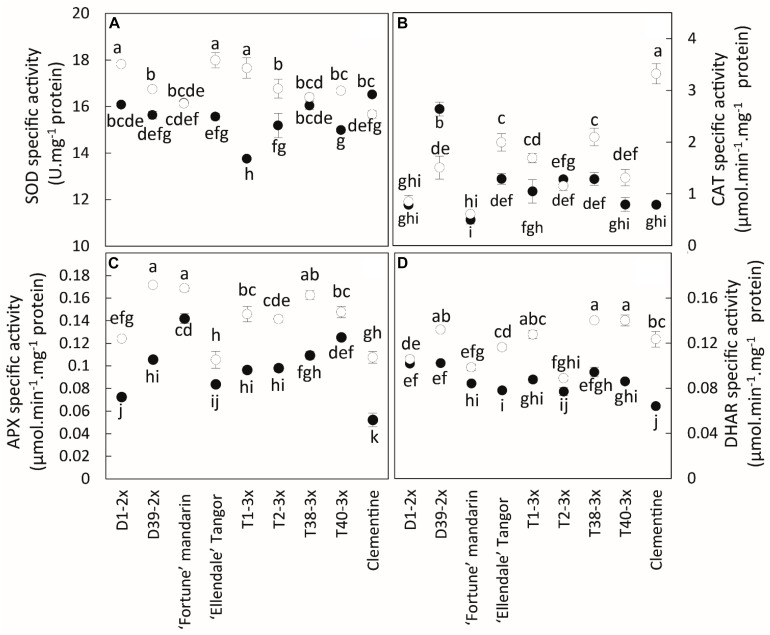
Comparison of antioxidant activities between the nine citrus varieties during warm period (black point) and cold period (white point). The specific activities were assayed for **(A)** SOD, **(B)** CAT, **(C)** APX, and **(D)** DHAR. Data are mean values ± (SE) of 4 independent biological replicates for each genotype (*n* = 4) obtained by pooling 15 fully expanded leaves. Data were analyzed using ANOVA and Fisher LSD testes (*P* < 0.05). Different lowercase letters indicate significant differences between the varieties and the sampling periods.

Overall, low temperatures increased the activity of antioxidant enzymes in all genotypes (Figure 6). No clear difference was found between triploid and other genotypes, whereas significant differences were found between some lines. The components of antioxidant systems were highly variable between the different varieties. Diploid and triploid lines could not be distinguished on these parameters.

### PCA in Leaves of Citrus Varieties Under Chilling Temperatures

**FIGURE 7:**
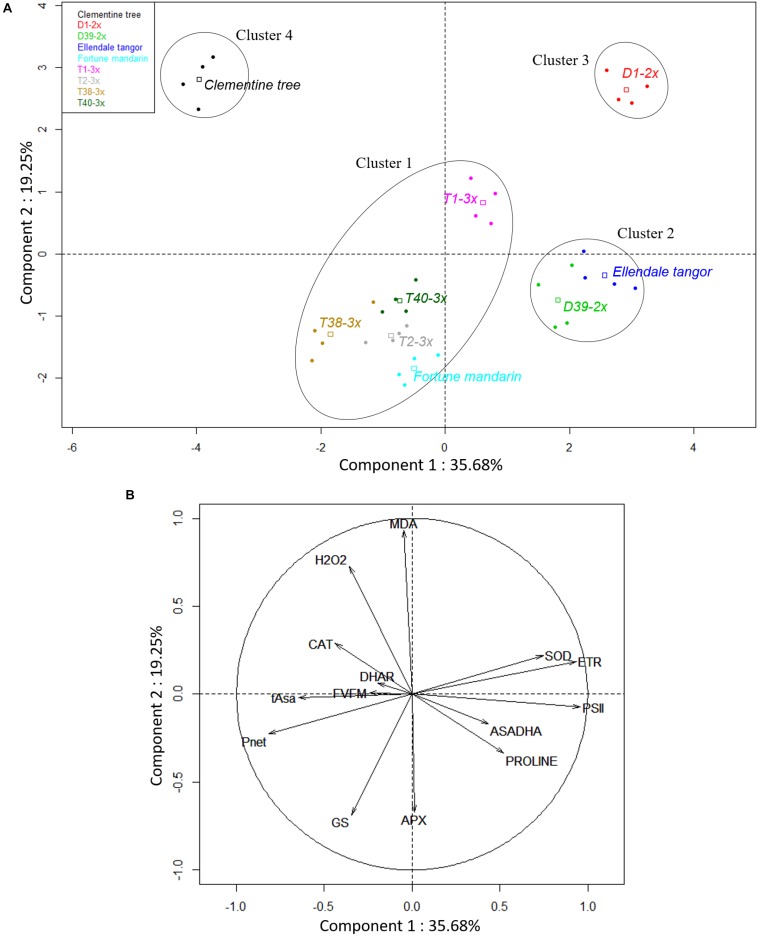
Biplot obtained from PCA performed on leaves of nine citrus varieties during cold period. **(A)** Repartition of varieties on the two first axes and **(B)** contribution of each physiological and biochemical variable to the two first axes of PCA. (PSII: *Φ_PSII_*).

To understand the differences between diploid and triploid lines during the cold period, PCA was performed with all parameters analyzed under this stressful condition (Figure 7). The first two principal components described 54.93% of the total variance in the population. Genotypes had clear differences in response to natural low temperatures. Antioxidant enzymes (SOD) and chlorophyll fluorescence parameters (ETR and *Φ_PSII_*) were the main parameters included in component 1. This component was also positively correlated with antioxidant molecules (Asa/DHA ratio and proline) and negatively correlated with *P*_net_ and tAsa content. Component 2 was positively correlated with oxidative markers (MDA and H_2_O_2_) while it was negatively correlated with APX and *g*_s_ (Figure 7B). Four clusters were clearly identified (Figure 7A). Component 1 separated cluster 1 (Fortune mandarin, T1-3X, T2-3X, T38-3x, and T40-3x) from cluster 2 (Ellendale tangor and D39-2x) and 3 (D1-2x), while component 2 separated cluster 1 and 2 from cluster 3 and 4 (clementine). Thus, Fortune mandarin and the four triploid hybrids were differentiated from diploid varieties by their enhanced photosynthetic capacities (high *P*_net_, *g*_s_ and *F_v_/F_m_* ratio). Triploid hybrids were also characterized by enhanced antioxidant enzymatic defenses (APX, DHAR and CAT) and higher tAsa content than diploid hybrids (clusters 2 and 3). Interestingly, D1-2x (cluster 3) was differentiated from Clementine (cluster 4) by higher SOD activity, ETR, and *Φ_PSII_*, while both clusters had increased oxidative marker accumulation.

### Differences in Leaf EO Composition in Citrus Varieties

**FIGURE 8:**
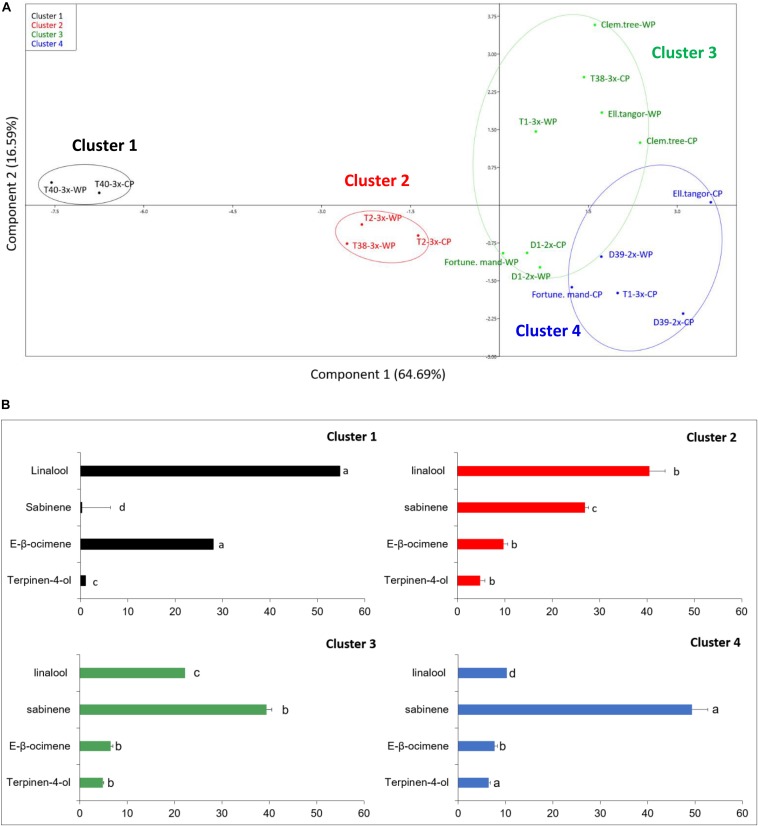
**(A)** Principal component analysis based on the chemical composition of leaves of nine citrus varieties during cold period (CP) and warm period (WP). **(B)** Comparison of chemotype from each cluster. Data are percentage of the total major compounds levels obtained from 3 independent biological replicates for each genotype (*n* = 3). The relative percentage of four major compounds was presented for each cluster. Different lowercase letters indicate significant differences between the clusters for each major compound.

Oil samples were submitted to GC-FID and GC/MS analyses. In total, 59 compounds were identified (Supplementary Table S1), including various monoterpenes accounting for 97.2–100% of the total EO chemical composition. Samples from the nine varieties harvested in both periods were submitted to statistical analyses along with 15 terpenes that exhibited a mean value higher than 0.1%. The data obtained for the EO chemical composition was submitted to a centered and scaled PCA. The coordinates of the individuals were analyzed by discriminant analysis in order to identify a structure among the genotypes based on their chemical composition. Hierarchical clustering dendrogram (Figure 8A) suggested the existence of two major groups (Supplementary Figure S2). These two main groups can each be subdivided into two sub-groups resulting in a total of four clusters based on the number of major components (Figure 8). Group I was characterized by 3x varieties except T1-3x while group II contained both parents, 2x varieties, clementine, and T1-3x variety.

To confirm the cluster analysis, the major compounds found in the varieties were included in a PCA analysis associated to the cluster analysis (Figure 8A). Four compounds were identified as being the most discriminant based on their contribution on the two discriminant axes: linalool, *E*-β-ocimene, sabinene, and terpinen-4-ol. Thus, PCA analysis representing 81.28% of the data variance showed four discriminant clusters (Figure 8A). The first axis separated the two principal groups defined by the hierarchical clustering: group 1 (composed of clusters 1 and 2) from group 2 (composed of clusters 3 and 4). The second axis separated the two principal groups into two sub-groups (cluster 1 *vs*. 2 and 3 *vs*. 4, respectively). No clear differences were observed between cold and warm periods, but discriminant analysis revealed differences between varieties. Cluster 1 was composed of only one triploid hybrid (T40-3x) and was characterized by an *E*-β-ocimene/linalool chemotype, exhibiting the highest amount of linalool (54.7%) and the lowest amount of sabinene (1.3%) which distinguished it from other clusters (Figure 8B). Cluster 2 was characterized by a linalool/sabinene chemotype while clusters 3 and 4 were characterized by a sabinene/linalool chemotype. No specific compounds discriminated the other clusters, but their overall chemical profiles differed with the proportion of major compounds. Cluster 2 contained T2-3x and T38-3x (warm period) and was characterized by a high amount of linalool (40.5%). A greater proportion of sabinene was found in clusters 3 and 4 (39.4 and 49.4% respectively). These clusters contained 2x varieties, both parents. T1-3x and T38-3x (cold period) were also found in these clusters.

## Discussion

### Comparison of Photosynthetic Responses to Natural Chilling Conditions Between 3x and 2x Varieties

The cold period was characterized by high levels of oxidative stress, antioxidant defenses, and lower photosynthetic activity. It has been reported that stomatal closure, illustrated by the decline in *g*_s_, was the first step followed by a decrease in Rubisco activity and then reduction of photosynthetic rate (Baker et al., 2007; Chaves et al., 2009). The higher values of *P*_net_ and *g*_s_ in 3x varieties argue for their lower sensitivity to low temperatures than 2x varieties. Polyploidization is known to increase stomatal size and decrease stomatal density (Masterson, 1994). Although Franks and Beerling (2009) showed that great density and small stomata were the only way to obtain the highest *g*_s_ maximum values, our results suggested that polyploidy may not necessarily induce reduced gas exchange and could be a good alternative to limiting the decrease in gas exchange, and thus, enhance tolerance to abiotic stresses. The chlorophyll fluorescence parameters provide insights on the photosynthesis properties under environmental constraints. Under optimal conditions, the *F_v_/F_m_* ratio reaches values about 0.83 in most C3 plants, as do our citrus trees during the warm period (Urban et al., 2017). The decrease in *F_v_/F_m_* we observed during the cold period is usually interpreted as being related to photo-inhibition (Genty et al., 1989). However, this decrease can also be due to the development of slowly relaxing quenching process, also known as non-photochemical quenching (Baker and Rosenqvist, 2004). Adams et al. (1995) also argue that induction of the photoprotective energy dissipation process can quite possibly account for the photoinhibition observed during winter stress. Thus, a decrease in *F_v_/F_m_* could be also interpreted in terms of a mechanism of photoprotection (Urban et al., 2017). Recently, several studies have monitored other indicators of photosynthetic performance, such as *Φ_PSII_* or electron transport rate (ETR), providing rapid information on PSII operating efficiency. Indeed, these parameters depend both on the efficiency of the absorbed energy donated to reaction centers and the rate of open reaction centers undergoing charge separation (Krall and Edwards, 1992; Baker et al., 2007). The natural chilling conditions (Figure 3A) seem to induce photo-inhibition of the photosynthetic apparatus (Baker et al., 2007), as expected (Allen and Ort, 2001; Santini et al., 2013; Oustric et al., 2017), but the 3x varieties would be less affected. Unfortunately, there is little information regarding the adaptation of 3x genotypes to abiotic stresses (Lu et al., 2009) while the better tolerance of tetraploid genotypes has often been observed, particularly among Citrus species (Hussain et al., 2012; Ruiz et al., 2016; Vieira et al., 2016; Oustric et al., 2019). Oustric et al. (2017) reported that the common clementine grafted on tetraploid rootstock had better photosynthetic capacity during natural chilling stress. Allario et al. (2013) concluded that tetraploid Rangpur lime rootstock conferred increased photosynthetic capacity to the diploid sweet orange scion when subjected to water deficit. In our study, 3x hybrids and clementine had different photochemical behaviors in response to low temperatures. The slow decline in *Φ_PSII_* and ETR associated with a weaker photo-inhibition suggests that electron flux through PSII was probably less affected. Triploid hybrids (except T1-3x) and clementine lose their excess energy as heat instead of being used to drive photosynthesis (Ribeiro et al., 2009). The consistent ETR/*P*_net_ ratio (close to the reference value ∼4) in 3x hybrids may suggest photoprotective energy dissipation activity by heat associated to NPQ (Yamori, 2016). Moreover, Azzabi et al. (2012) reported that thermal dissipation within photosystems acts as a safety strategy for cutting down on excess light energy and limiting ROS generation. Machado et al. (2013) also argued that NPQ increase was effective at preventing excessive energy pressure at PSII in Valencia sweet orange grafted on Swingle citrumelo subjected to night-time chilling temperatures. Some studies reported a negative correlation between *Φ_PSII_* and NPQ. Thus, a decrease in *Φ_PSII_* could mean an increase in thermal dissipation (Maxwell and Johnson, 2000; Ribeiro et al., 2009). The NPQ mechanism is characterized by the pH-dependent xanthophyll cycle which provides effective protection of reaction centers at low temperatures and high light (Baker et al., 2007). High values of *Φ_PSII_* and ETR associated with the sharp decline in *F_v_/F_m_* in 2x hybrids suggest an involvement of electron sinks other than CO_2_. ETR/*P*_net_ has been largely recognized as an indicator of increased photorespiration (Flexas et al., 1999; Ribeiro et al., 2009; Machado et al., 2013). The high values of ETR/*P*_net_ (more than the theoretical value ∼4) for 2x hybrids support the contribution of alternative pathways. According to Baker et al. (2007), the proportion of electrons driven to the alternative pathway increases under stress conditions. Thus, photorespiration and the Mehler reaction are the main alternative sinks during environmental constraints (Fryer et al., 1998; Allen and Ort, 2001). Since photorespiration is sensitive to low temperatures, the Mehler reaction would be the main way to dissipate excess energy (Fryer et al., 1998; Flexas et al., 1999). Increases in alternative electron sinks have already been reported in 2x citrus (Medina et al., 2002; Ribeiro et al., 2009; Machado et al., 2010; Santos et al., 2011). Machado et al. (2013) have found a decline in photosynthetic parameters (*P*_net_, *g*_s_, *Φ_PSII_*) associated with an increase in ETR/*P*_net_ ratio in Valencia sweet orange scion, under cold conditions. The alternative pathways would not be sufficient for preventing photo-inhibition in 2x hybrids. The involvement of O_2_-consuming processes induced ROS production, followed by a higher up-regulation of antioxidant mechanisms.

### Relevant Traits Discriminating 2x and 3x Varieties for Chilling Sensitivity

The 3x varieties and their parent Fortune mandarin appear to have less marked photo-inhibition, as supported by their great photosynthetic parameters (*P*_net_, *g*_s_ and *F_v_/F_m_*) (Figure 7B). The ROS induced by photoprotective processes can be scavenged by effective antioxidant defenses. In our study, specific activities of SOD, APX, and DHAR were enhanced at low temperatures, indicating that an effective antioxidant response is implemented to limit oxidative damage (Figure 6). The greater SOD and APX activities under low temperatures contribute to protecting the cells by removing superoxide anion and H_2_O_2_ (Mittler, 2002). High SOD and APX specific activities were also found in clementine grafted in tetraploid citrus seedlings under natural chilling stress (Oustric et al., 2019) and in citrumelo and Rangpur lime citrus rootstock grafted with sweet orange during low temperatures (Machado et al., 2013). The increased DHAR activity may be helpful for ascorbate regeneration resulting from an increase in the Asa/DHA ratio in response to low temperatures. The differences in antioxidants between 3x varieties suggest different responses between the lines and may be related to the subcellular location of the enzymes. Identifying the respective enzyme isoforms will provide information on their location and, thus, on the antioxidant mechanism. The high total ascorbate (tAsa) content in certain triploid lines, mandarin Fortune, and clementine (Figure 5A) evidence *de novo* ascorbate synthesis under natural low temperatures, in addition to the regeneration of reduced ascorbate by DHAR. Previous studies have reported that ascorbate can play a significant role in thermal dissipation of excess energy, acting as a co-factor for violaxanthin de-epoxidase to produce zeaxanthin (Saga et al., 2010; Smirnoff, 2018). Jahns et al. (2009) also argued that the increase in ascorbate results in an increase in NPQ and zeaxanthin formation. Therefore, the ascorbate would help in heat dissipation and, thus, in limiting excess electron flux at PSII in 3x lines (Figures 3B,D).

Cluster 2 contained D39-2x and its parent Ellendale tangor while the D1-2x variety belonged to cluster 3. Both clusters were differentiated from the other clusters mainly by ETR and *Φ_PSII_*. Associated with low values in photosynthetic parameters, it suggests the involvement of an alternative electron sink leading to ROS generation, such as superoxide anion (Genty et al., 1989; Baker, 2008). Consistent with these data, low temperatures induced accumulation of H_2_O_2_ (Figure 4A) and increased SOD activity (Figure 6A). Cluster 3 was also differentiated from cluster 2 by high oxidative damage (Figure 7), indicating that D1-2x was more sensitive to chilling temperatures than D39-2x. These findings were consistent with the previous ranking by MDA levels (Supplementary Figure S1).

Clementine (cluster 4) exhibited contrasting behaviors compared to the other varieties. It was differentiated by higher oxidative marker accumulation (H_2_O_2_ and MDA) than clusters 1 and 2 (Figure 7A). Despite the higher photosynthetic rate and CAT and DHAR activities, it appeared to be more sensitive to low temperatures than 3x hybrids (Figure 4). Some studies have already reported that cold temperatures disturb photosynthesis performance and increase oxidative damage (Machado et al., 2013; Santini et al., 2013; Oustric et al., 2019). Proline accumulates in cell plants in response to many environmental stresses, like osmotic stress and drought. Some studies have reported that proline accumulation was associated with H_2_O_2_, MDA decline, or lipid peroxidation (de Campos et al., 2011). This negative correlation was also observed in our study (Figure 1). Our results showed that triploidy enhance photosynthetic activities likely by limiting photo-inhibition, whereas the oxidative metabolism would not be sufficient to clearly discriminate triploid from diploid lines under stressful conditions. The complexity of oxidative metabolism and its cellular compartmentalization hinders the understanding of its contribution under stress conditions.

Citrus species are a major source of VOCs and their variations are mostly temperature dependent (Fares et al., 2012). However, in our orchard conditions, the chemical composition of the varieties was not significantly different between the study periods. In plants, VOCs are involved in various functions, such as defense and reproduction. Few studies reported changes in VOC content in Citrus varieties as a consequence of environmental stresses. Some terpenes (sabinene, linalool, *E*-β-ocimene) were shown to be involved in plant adaptation to drought (Vieira et al., 2016), winter flooding, and salinity (Velikova et al., 2012). Loreto et al. (1998) described the protective roles of VOCs, especially mono- and sesquiterpenes (sabinene, *E*-β-ocimene), under heat stress. Thus, while β-ocimene appeared to be insensitive to high temperatures, increased emission in other monoterpenes such as sabinene were reported in response to high temperatures. The authors also suggested a relationship between monoterpene emission, photosynthetic performance, and oxidative stress. It was discovered that monoterpene fumigation stimulates photosynthesis and decreases ROS levels and cell damage (Loreto and Schnitzler, 2010). Monoterpene emission had been observed to vary under other abiotic stresses. For example, inhibition of linalool emission was reported during drought stress (Gouinguené and Turlings, 2002).

The chemical composition of leaf EOs have already been studied in several citrus species, underlying a great variability among the citrus cultivars (Lota et al., 2001; Fanciullino et al., 2006; Espina et al., 2011; Santos et al., 2015). The most common chemotypes were sabinene/linalool and γ-terpinene/linalool (Lota et al., 2001). The varieties we studied can be distinguished into four clusters for leaf EO (Figure 8A). It is interesting to note the T40-3x variety had the greatest proportion of *E*-β-ocimene among all leaf EOs of the Citrus species studied (Lota et al., 2001), confirming the wide diversity of VOCs profiles among Citrus species. Chen et al. (2008) revealed that β-ocimene emission was also stimulated under oxidative stress induced by nitrogen deficiency. Even if no variation of *E*-β-ocimene was found for the T40-3x variety between warm and cold periods, we assume that its high content could help to limit oxidative stress as indicated by the low cellular damage in this variety (Figure 4). The large linalool proportion found in 3x varieties (Figure 8B) was previously found in mandarin leaf EOs from Citrus *reticulata* Blanco (Lota et al., 2001). Overall, our results suggested the VOC profile was mainly influenced by the ploidy level.

More globally, it is interesting to note that up or downregulation of interest genes that occur only in polyploid lines could be beneficial for its stress tolerance. For example, Del Pozo and Ramirez-Parra (2014) found that gene expression of some transcription factors (TFs) are over-represented between the tetraploidy target genes such as WRKY, DREB, and ERF transcription factors, which appear to play a crucial role in tolerance to cold, drought, and salt stress (Chen et al., 2012; Wang et al., 2017). Higher expression of transcripts of genes encoding antioxidant enzymes (CAT, APX and glutathione reductase) had also been reported in tetraploid lines under water deficit, compared to the diploid counterparts (Yan et al., 2019).

## Conclusion

This study was the first to look at the impact of natural chilling temperatures combined with triploidy level in new citrus species. Our analysis suggested that triploidy may improve photosynthetic performances under chilling temperatures. The small decrease in PSII photochemistry and the maintenance of ETR/*P*_net_ ratio indicated effective photo-protective mechanisms to counteract toxic effects induced by low temperatures, suggesting 3x varieties have a high tolerance to low temperatures. The antioxidant response does not allow us to discriminate triploid varieties from others and explain enhanced photosynthetic performances in 3x varieties. Chemical analysis uncovered atypical leaf VOC profiles that can be related to ploidy level. Triploid varieties had the highest proportions of *E*-β-ocimene, linalool, and terpenes involved in oxidative stress protection. To deepen our study, it would be interesting to collect biomass data, including investigation on fruit yield and nutrient uptake behavior. The use of triploidy appear to be a relevant breeding strategy to improve the fresh citrus fruit market and to develop new seedless citrus commercial varieties that have enhanced abiotic stress tolerance.

## Data Availability Statement

The datasets generated for this study are available on request to the corresponding author.

## Author Contributions

RL collected the test data, performed the statistical analyses, interpreted the results, and drafted the manuscript. JS interpreted the results and drafted the manuscript. FT and MG collected the chemical data. MG helped to perform statistical analyses. LB, JG, SH, YF, and RM helped draft the manuscript.

## Conflict of Interest

The authors declare that the research was conducted in the absence of any commercial or financial relationships that could be construed as a potential conflict of interest.

## Footnotes

1 http://www.R-project.org

## References

[B1] Adams W. I. Demmig-Adams B. Verhoeven A. S. Barker D. H. (1995). Photoinhibition” during winter stress: involvement of sustained xanthophyll cycle-dependent energy dissipation. *Funct. Plant Biol.* 22 261–276. 10.1071/pp9950261

[B2] Agurla S. Gahir S. Munemasa S. Murata Y. Raghavendra A. S. (2018). “Mechanism of stomatal closure in plants exposed to drought and cold stress,” in *Survival Strategies in Extreme Cold and Desiccation: Adaptation Mechanisms and Their Applications*, eds Iwaya-Inoue M. Sakurai M. Uemura M. (Singapore: Springer), 215–232. 10.1007/978-981-13-1244-1_12 30288712

[B3] Aleza P. Juárez J. Cuenca J. Ollitrault P. Navarro L. (2012). Extensive *Citrus* triploid hybrid production by 2x × 4x sexual hybridizations and parent-effect on the length of the juvenile phase. *Plant Cell Rep.* 31 1723–1735. 10.1007/s00299-012-1286-0 22614256

[B4] Aleza P. Juárez J. Ollitrault P. Navarro L. (2010). Polyembryony in non-apomictic *Citrus* genotypes. *Ann. Bot.* 106 533–545. 10.1093/aob/mcq148 20675656 PMC2944972

[B5] Allario T. Brumos J. Colmenero-Flores J. M. Iglesias D. J. Pina J. A. Navarro L. (2013). Tetraploid Rangpur lime rootstock increases drought tolerance via enhanced constitutive root abscisic acid production. *Plant Cell Environ.* 36 856–868. 10.1111/pce.12021 23050986

[B6] Allario T. Brumos J. Colmenero-Flores J. M. Tadeo F. Froelicher Y. Talon M. (2011). Large changes in anatomy and physiology between diploid Rangpur lime (*Citrus limonia*) and its autotetraploid are not associated with large changes in leaf gene expression. *J. Exp. Bot.* 62 2507–2519. 10.1093/jxb/erq467 21273338

[B7] Allen D. J. Ort D. R. (2001). Impacts of chilling temperatures on photosynthesis in warm-climate plants. *Trends Plant Sci.* 6 36–42. 10.1016/s1360-1385(00)01808-2 11164376

[B8] Azzabi G. Pinnola A. Betterle N. Bassi R. Alboresi A. (2012). Enhancement of non-photochemical quenching in the bryophyte *Physcomitrella patens* during acclimation to salt and osmotic stress. *Plant Cell Physiol.* 53 1815–1825. 10.1093/pcp/pcs124 22952250

[B9] Baker N. R. (2008). Chlorophyll fluorescence: a probe of photosynthesis in vivo. *Annu. Rev. Plant Biol.* 59 89–113. 10.1146/annurev.arplant.59.032607.092759 18444897

[B10] Baker N. R. Harbinson J. Kramer D. M. (2007). Determining the limitations and regulation of photosynthetic energy transduction in leaves. *Plant Cell Environ.* 30 1107–1125. 10.1111/j.1365-3040.2007.01680.x 17661750

[B11] Baker N. R. Rosenqvist E. (2004). Applications of chlorophyll fluorescence can improve crop production strategies: an examination of future possibilities. *J. Exp. Bot.* 55 1607–1621. 10.1093/jxb/erh196 15258166

[B12] Bradford M. M. (1976). A rapid and sensitive method for the quantitation of microgram quantities of protein utilizing the principle of protein-dye binding. *Anal. Biochem.* 72 248–254. 10.1016/0003-2697(76)90527-3 942051

[B13] Carillo P. Gibon Y. (2011). *PROTOCOL: Extraction and Determination of Proline*. PrometheusWiki.

[B14] Chaves M. M. Flexas J. Pinheiro C. (2009). Photosynthesis under drought and salt stress: regulation mechanisms from whole plant to cell. *Ann. Bot.* 103 551–560. 10.1093/aob/mcn125 18662937 PMC2707345

[B15] Chen L. Song Y. Li S. Zhang L. Zou C. Yu D. (2012). The role of WRKY transcription factors in plant abiotic stresses. *Biochim. Biophys. Acta* 1819 120–128. 10.1016/j.bbagrm.2011.09.002 21964328

[B16] Chen Y. Schmelz E. A. Wäckers F. Ruberson J. R. (2008). Cotton plant, *Gossypium hirsutum* L., defense in response to nitrogen fertilization. *J. Chem. Ecol.* 34 1553–1564. 10.1007/s10886-008-9560-x 19020938

[B17] Chen Z. J. (2007). Genetic and epigenetic mechanisms for gene expression and phenotypic variation in plant polyploids. *Annu. Rev. Plant Biol.* 58 377–406. 10.1146/annurev.arplant.58.032806.103835 17280525 PMC1949485

[B18] Copolovici L. Kännaste A. Pazouki L. Niinemets Ü (2012). Emissions of green leaf volatiles and terpenoids from *Solanum lycopersicum* are quantitatively related to the severity of cold and heat shock treatments. *J. Plant Physiol.* 169 664–672. 10.1016/j.jplph.2011.12.019 22341571

[B19] Costa S. N. Cortez P. A. da Hora Almeida L. A. Martins F. M. Soares Filho W. Filho M. A. C. (2019). Triploid frequency of sexual hybridization and pollen and ovary development in mandarins. *Braz. J. Bot.* 42 73–82. 10.1007/s40415-019-00513-6

[B20] de Campos M. K. F. de Carvalho K. de Souza F. S. Marur C. J. Pereira L. F. P. Filho J. C. B. (2011). Drought tolerance and antioxidant enzymatic activity in transgenic ‘Swingle’ citrumelo plants over-accumulating proline. *Environ. Exp. Bot.* 72 242–250. 10.1016/j.envexpbot.2011.03.009

[B21] Del Pozo J. C. Ramirez-Parra E. (2014). Deciphering the molecular basis for drought tolerance in *Arabidopsis* autotetraploids. *Plant Cell Environ.* 37 2722–2737. 10.1111/pce.12344 24716850

[B22] Esen A. Soost R. K. (1971). Unexpected triploids in *Citrus*: their origin, identification, and possible use. *J. Hered.* 62 329–333. 10.1093/oxfordjournals.jhered.a108186

[B23] Esen A. Soost R. K. Geraci G. (1978). Seed set, size, and development after 4x × 2x and 4x × 4x crosses in *Citrus*. *Euphytica* 27 283–294. 10.1007/BF00039144

[B24] Espina L. Somolinos M. Lorán S. Conchello P. García D. Pagán R. (2011). Chemical composition of commercial *Citrus* fruit essential oils and evaluation of their antimicrobial activity acting alone or in combined processes. *Food Control* 22 896–902. 10.1016/j.foodcont.2010.11.021

[B25] Fanciullino A.-L. Tomi F. Luro F. Desjobert J. M. Casanova J. (2006). Chemical variability of peel and leaf oils of mandarins. *Flavour Fragr. J.* 21 359–367. 10.1002/ffj.1658 11068126

[B26] FAO (2017). *Citrus Fruit, Fresh and Processed - Statistical Bulletin*. Available online at: http://www.fao.org/economic/est/est-commodities/citrus-fruit/en/

[B27] Fares S. Park J.-H. Gentner D. R. Weber R. Ormeño E. Karlik J. (2012). Seasonal cycles of biogenic volatile organic compound fluxes and concentrations in a California *Citrus* orchard. *Atmos. Chem. Phys. Discuss.* 12 17987–18027. 10.5194/acpd-12-17987-2012

[B28] Flexas J. Badger M. Chow W. S. Medrano H. Osmond C. B. (1999). Analysis of the relative increase in photosynthetic O2 uptake when photosynthesis in grapevine leaves is inhibited following low night temperatures and/or water stress. *Plant Physiol.* 121 675–684. 10.1104/pp.121.2.675 10517860 PMC59431

[B29] Franks P. J. Beerling D. J. (2009). Maximum leaf conductance driven by CO2 effects on stomatal size and density over geologic time. *Proc. Natl. Acad. Sci. U.S.A.* 106 10343–10347. 10.1073/pnas.0904209106 19506250 PMC2693183

[B30] Fryer M. J. Andrews J. R. Oxborough K. Blowers D. A. Baker N. R. (1998). Relationship between CO_2_ assimilation, photosynthetic electron transport, and active O_2_ metabolism in leaves of maize in the field during periods of low temperature. *Plant Physiol.* 116:571. 10.1104/pp.116.2.571 9490760 PMC35114

[B31] Genty B. Briantais J.-M. Baker N. R. (1989). The relationship between the quantum yield of photosynthetic electron transport and quenching of chlorophyll fluorescence. *Biochim. Biophys. Acta* 990 87–92. 10.1016/S0304-4165(89)80016-9

[B32] Gill S. S. Tuteja N. (2010). Reactive oxygen species and antioxidant machinery in abiotic stress tolerance in crop plants. *Plant Physiol. Biochem.* 48 909–930. 10.1016/j.plaphy.2010.08.016 20870416

[B33] Gouinguené S. P. Turlings T. C. J. (2002). The effects of abiotic factors on induced volatile emissions in corn plants. *Plant Physiol.* 129 1296–1307. 10.1104/pp.001941 12114583 PMC166523

[B34] Guenther A. Karl T. Harley P. Wiedinmyer C. Palmer P. I. Geron C. (2006). Estimates of global terrestrial isoprene emissions using MEGAN (model of emissions of gases and aerosols from nature). *Atmos. Chem. Phys.* 6 3181–3210. 10.5194/acp-6-3181-2006

[B35] Hijaz F. Nehela Y. Killiny N. (2016). Possible role of plant volatiles in tolerance against huanglongbing in citrus. *Plant Signal. Behav.* 11:e1138193. 10.1080/15592324.2016.1138193 26829496 PMC4883952

[B36] Hodges D. M. DeLong J. M. Forney C. F. Prange R. K. (1999). Improving the thiobarbituric acid-reactive-substances assay for estimating lipid peroxidation in plant tissues containing anthocyanin and other interfering compounds. *Planta* 207 604–611. 10.1007/s004250050524 28456836

[B37] Hoshino Y. Miyashita T. Thomas T. D. (2011). In vitro culture of endosperm and its application in plant breeding: approaches to polyploidy breeding. *Sci. Horticult.* 130 1–8. 10.1016/j.scienta.2011.06.041

[B38] Hussain H. A. Hussain S. Khaliq A. Ashraf U. Anjum S. A. Men S. (2018). Chilling and drought stresses in crop plants: implications, cross talk, and potential management opportunities. *Front. Plant Sci.* 9:393. 10.3389/fpls.2018.00393 29692787 PMC5902779

[B39] Hussain S. Curk F. Dhuique-Mayer C. Urban L. Ollitrault P. Luro F. (2012). Autotetraploid trifoliate orange (*Poncirus trifoliata*) rootstocks do not impact clementine quality but reduce fruit yields and highly modify rootstock/scion physiology. *Sci. Horticult.* 134 100–107. 10.1016/j.scienta.2011.11.008

[B40] Jahns P. Latowski D. Strzalka K. (2009). Mechanism and regulation of the violaxanthin cycle: the role of antenna proteins and membrane lipids. *Biochim. Biophys. Acta* 1787 3–14. 10.1016/j.bbabio.2008.09.013 18976630

[B41] Jiang Z. Y. Woollard A. C. Wolff S. P. (1991). Lipid hydroperoxide measurement by oxidation of Fe2+ in the presence of xylenol orange. Comparison with the TBA assay and an iodometric method. *Lipids* 26 853–856. 10.1007/bf02536169 1795606

[B42] Krall J. P. Edwards G. E. (1992). Relationship between photosystem II activity and CO2 fixation in leaves. *Physiol. Plant.* 86 180–187. 10.1111/j.1399-3054.1992.tb01328.x

[B43] Krug C. A. (1943). Chromosome numbers in the subfamily aurantioideae with special reference to the genus *Citrus*. *Bot. Gaz.* 104 602–611. 10.1086/335173

[B44] Le S. Josse J. Husson F. (2008). FactoMineR: an R package for multivariate analysis. *J. Stat. Softw.* 25 1–18. 10.18637/jss.v025.i01

[B45] Lee L. (1988). Citrus polyploidy – origins and potential for cultivar improvement. *Aust. J. Agric. Res.* 39 735–747.

[B46] Loreto F. Förster A. Dürr M. Csiky O. Seufert G. (1998). On the monoterpene emission under heat stress and on the increased thermotolerance of leaves of *Quercus ilex* L. fumigated with selected monoterpenes. *Plant Cell Environ.* 21 101–107. 10.1046/j.1365-3040.1998.00268.x

[B47] Loreto F. Schnitzler J.-P. (2010). Abiotic stresses and induced BVOCs. *Trends Plant Sci.* 15 154–166. 10.1016/j.tplants.2009.12.006 20133178

[B48] Lota D. Tomi F. Casanova J. C. (2001). Chemical variability of peel and leaf essential oils of 15 species of mandarins. *Biochem. Syst. Ecol.* 29 77–104. 10.1016/S0305-1978(00)00029-6 11068126

[B49] Lu S. Chen C. Wang Z. Guo Z. Li H. (2009). Physiological responses of somaclonal variants of triploid bermudagrass (*Cynodon transvaalensis* × *Cynodon dactylon*) to drought stress. *Plant Cell Rep.* 28 517–526. 10.1007/s00299-008-0649-z 19050896

[B50] Machado D. F. S. P. Machado E. C. Machado R. S. Ribeiro R. V. (2010). Effects of low night temperature and rootstocks on diurnal variation of leaf gas exchange rates and photochemical activity of “Valência” sweet orange plants. *Revist. Bras. Fruticult.* 32 351–359. 10.1590/S0100-29452010005000064

[B51] Machado D. F. S. P. Ribeiro R. V. Silveira J. A. G. Magalhães Filho J. R. Machado E. C. (2013). Rootstocks induce contrasting photosynthetic responses of orange plants to low night temperature without affecting the antioxidant metabolism. *Theor. Exp. Plant Physiol.* 25 26–35. 10.1590/S2197-00252013000100004

[B52] Madlung A. (2013). Polyploidy and its effect on evolutionary success: old questions revisited with new tools. *Heredity* 110 99–104. 10.1038/hdy.2012.79 23149459 PMC3554449

[B53] Masterson J. (1994). Stomatal size in fossil plants: evidence for polyploidy in majority of angiosperms. *Science* 264 421–424. 10.1126/science.264.5157.421 17836906

[B54] Maxwell K. Johnson G. N. (2000). Chlorophyll fluorescence–a practical guide. *J. Exp. Bot.* 51 659–668. 10.1093/jexbot/51.345.659 10938857

[B55] Medina C. L. Souza R. P. Machado E. C. Ribeiro R. V. Silva J. A. B. (2002). Photosynthetic response of citrus grown under reflective aluminized polypropylene shading nets. *Sci. Horticult.* 96 115–125. 10.1016/S0304-4238(02)00085-7

[B56] Mittler R. (2002). Oxidative stress, antioxidants and stress tolerance. *Trends Plant Sci.* 7 405–410. 10.1016/S1360-1385(02)02312-9 12234732

[B57] Navarro L. Aleza P. Cuenca J. Juárez J. José A. Carmen Ortega P. (2015). The mandarin triploid breeding program in Spain. *Acta Horticulturae* 1065, 389–395. 10.17660/ActaHortic.2015.1065.48

[B58] Oustric J. Morillon R. Luro F. Herbette S. Lourkisti R. Giannettini J. (2017). Tetraploid carrizo citrange rootstock (*Citrus sinensis* Osb. × *Poncirus trifoliata* L. Raf.) enhances natural chilling stress tolerance of common clementine (*Citrus clementina* Hort. ex Tan). *J. Plant Physiol.* 214 108–115. 10.1016/j.jplph.2017.04.014 28478318

[B59] Oustric J. Morillon R. Luro F. Herbette S. Martin P. Giannettini J. (2019). Nutrient deficiency tolerance in *Citrus* is dependent on genotype or ploidy level. *Front. Plant Sci.* 10:127. 10.3389/fpls.2019.00127 30853962 PMC6396732

[B60] Peñuelas J. Staudt M. (2010). BVOCs and global change. *Trends Plant Sci.* 15 133–144. 10.1016/j.tplants.2009.12.005 20097116

[B61] Possell M. Loreto F. (2013). “The role of volatile organic compounds in plant resistance to abiotic stresses: responses and mechanisms,” in *Biology, Controls and Models of Tree Volatile Organic Compound Emissions*, eds Niinemets Ü Monson R. K. (Dordrecht: Springer Netherlands), 209–235. 10.1007/978-94-007-6606-8_8

[B62] Ramsey J. (2011). Polyploidy and ecological adaptation in wild yarrow. *Proc. Natl. Acad. Sci. U.S.A.* 108 7096–7101. 10.1073/pnas.1016631108 21402904 PMC3084070

[B63] Ribeiro R. V. Machado E. C. Santos M. G. Oliveira R. F. (2009). Seasonal and diurnal changes in photosynthetic limitation of young sweet orange trees. *Environ. Exp. Bot.* 66 203–211. 10.1016/j.envexpbot.2009.03.011

[B64] Rouiss H. Bakry F. Froelicher Y. Navarro L. Aleza P. Ollitrault P. (2018). Origin of *C. latifolia* and *C. aurantiifolia* triploid limes: the preferential disomic inheritance of doubled-diploid ‘Mexican’ lime is consistent with an interploid hybridization hypothesis. *Ann. Bot.* 121 571–585. 10.1093/aob/mcx179 29293884 PMC5838810

[B65] Ruiz M. Pensabene B. G. Quiñones A. García-Lor A. Morillon R. Ollitrault P. (2018). Molecular characterization and stress tolerance evaluation of new allotetraploid somatic hybrids between *Carrizo Citrange* and *Citrus macrophylla* W. rootstocks. *Front. Plant Sci.* 9:901. 10.3389/fpls.2018.00901 30123223 PMC6085489

[B66] Ruiz M. Quiñones A. Martínez-Cuenca M. R. Aleza P. Morillon R. Navarro L. (2016). Tetraploidy enhances the ability to exclude chloride from leaves in carrizo citrange seedlings. *J. Plant Physiol.* 205 1–10. 10.1016/j.jplph.2016.08.002 27589221

[B67] Saga G. Giorgetti A. Fufezan C. Giacometti G. M. Bassi R. Morosinotto T. (2010). Mutation analysis of violaxanthin de-epoxidase identifies substrate-binding sites and residues involved in catalysis. *J. Biol. Chem.* 285 23763–23770. 10.1074/jbc.M110.115097 20507981 PMC2911307

[B68] Saleh B. Allario T. Dambier D. Ollitrault P. Morillon R. (2008). Tetraploid *Citrus* rootstocks are more tolerant to salt stress than diploid. *C. R. Biol.* 331 703–710. 10.1016/j.crvi.2008.06.007 18722990

[B69] Santini J. Giannettini J. Pailly O. Herbette S. Ollitrault P. Berti L. (2013). Comparison of photosynthesis and antioxidant performance of several *Citrus* and *Fortunella* species (Rutaceae) under natural chilling stress. *Trees* 27 71–83. 10.1007/s00468-012-0769-5

[B70] Santos C. M. A. Ribeiro R. V. Magalhães Filho J. R. Machado D. F. S. P. Machado E. C. (2011). Low substrate temperature imposes higher limitation to photosynthesis of orange plants as compared to atmospheric chilling. *Photosynthetica* 49 546–554. 10.1007/s11099-011-0071-6

[B71] Santos J. Z. Almeida L. A. H. Soares Filho W. S. Bizzo H. R. Santos M. C. da S. (2015). Chemical characterization of the essential oils from leaves of mandarins Sunki, Cleopatra and their hybrids. *J. Essent. Oil Res.* 27 1–8. 10.1080/10412905.2014.973067

[B72] Smirnoff N. (2018). Ascorbic acid metabolism and functions: a comparison of plants and mammals. *Free Radic. Biol. Med.* 122 116–129. 10.1016/j.freeradbiomed.2018.03.033 29567393 PMC6191929

[B73] Stevens R. Page D. Gouble B. Garchery C. Zamir D. Causse M. (2008). Tomato fruit ascorbic acid content is linked with monodehydroascorbate reductase activity and tolerance to chilling stress. *Plant Cell Environ.* 31 1086–1096. 10.1111/j.1365-3040.2008.01824.x 18433441

[B74] Tholl D. (2015). “Biosynthesis and biological functions of terpenoids in plants,” in *Biotechnology of Isoprenoids*, eds Schrader J. Bohlmann J. (Basel: Springer International Publishing), 63–106. 10.1007/10_2014_295 25583224

[B75] Urban L. Aarrouf J. Bidel L. P. R. (2017). Assessing the effects of water deficit on photosynthesis using parameters derived from measurements of leaf gas exchange and of chlorophyll a fluorescence. *Front. Plant Sci.* 8:2068. 10.3389/fpls.2017.02068 29312367 PMC5735977

[B76] Velikova V. Mantia T. L. Lauteri M. Michelozzi M. Nogues I. Loreto F. (2012). The impact of winter flooding with saline water on foliar carbon uptake and the volatile fraction of leaves and fruits of lemon (*Citrus* × limon) trees. *Funct. Plant Biol.* 39 199–213. 10.1071/FP11231 32480774

[B77] Vickers C. E. Gershenzon J. Lerdau M. T. Loreto F. (2009). A unified mechanism of action for volatile isoprenoids in plant abiotic stress. *Nat. Chem. Biol.* 5 283–291. 10.1038/nchembio.158 19377454

[B78] Vieira D. D. S. S. Emiliani G. Michelozzi M. Centritto M. Luro F. Morillon R. (2016). Polyploidization alters constitutive content of volatile organic compounds (VOC) and improves membrane stability under water deficit in Volkamer lemon (*Citrus limonia* Osb.) leaves. *Environ. Exp. Bot.* 126 1–9. 10.1016/j.envexpbot.2016.02.010

[B79] Wang J. Tian L. Lee H.-S. Wei N. E. Jiang H. Watson B. (2006). Genomewide nonadditive gene regulation in *Arabidopsis* allotetraploids. *Genetics* 172 507–517. 10.1534/genetics.105.047894 16172500 PMC1456178

[B80] Wang K. Yin X.-R. Zhang B. Grierson D. Xu C.-J. Chen K.-S. (2017). Transcriptomic and metabolic analyses provide new insights into chilling injury in peach fruit. *Plant Cell Environ.* 40 1531–1551. 10.1111/pce.12951 28337785

[B81] Yamori W. (2016). Photosynthetic response to fluctuating environments and photoprotective strategies under abiotic stress. *J. Plant Res.* 129 379–395. 10.1007/s10265-016-0816-1 27023791

[B82] Yan L. Li Y. Dong Y. Fan G. (2019). Transcriptional and post-transcriptional responses of diploid and autotetraploid *Paulownia tomentosa*? × *?Paulownia fortunei* under water-deficit condition. *Braz. J. Bot.* 42 623–641. 10.1007/s40415-019-00566-7

