# Supplementary material for: Triploid Citrus Genotypes Have a Better Tolerance to Natural Chilling Conditions of Photosynthetic Capacities and Specific Leaf Volatile Organic Compounds
Source: Front Plant Sci. 2020 Apr 21;11:330. doi: 10.3389/fpls.2020.00330 (PMC7189121; doi:10.3389/fpls.2020.00330)
Supplement: Supplementary file 1 [file Data_Sheet_1.docx]

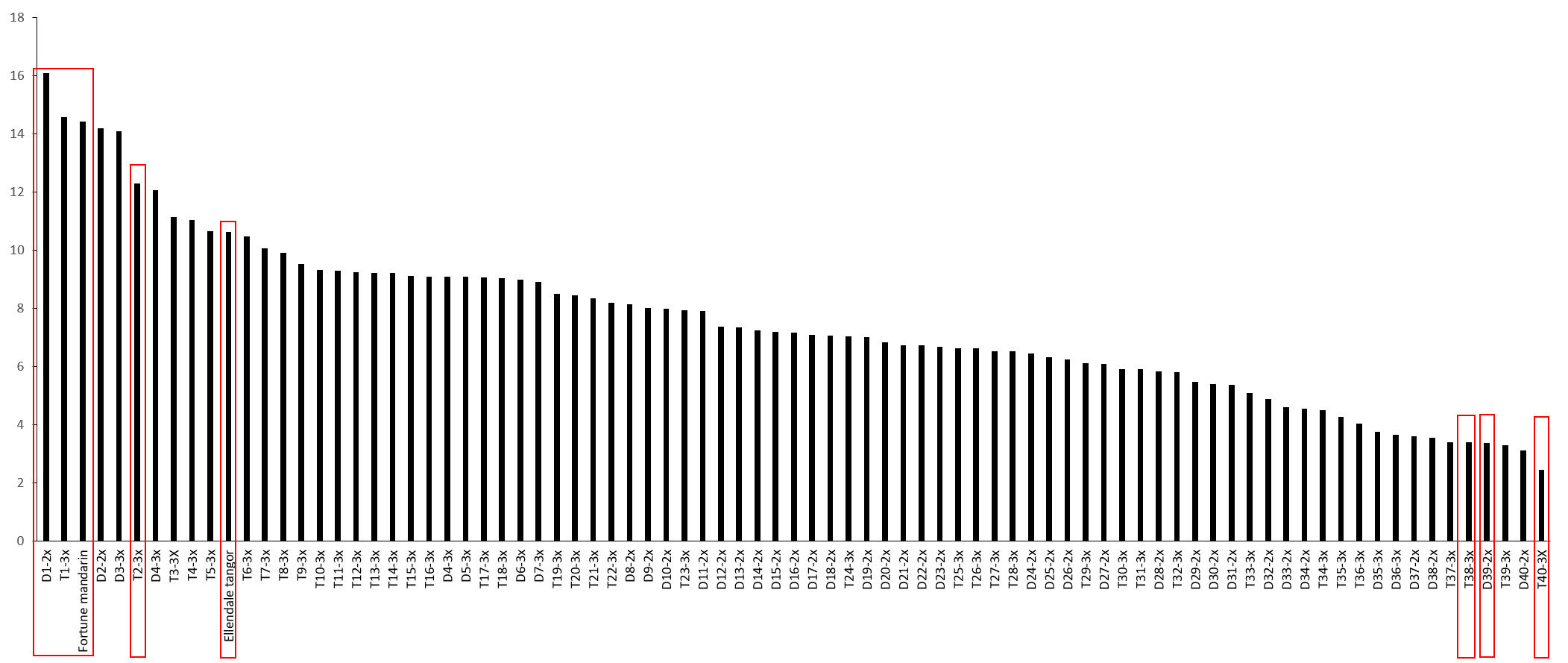


**Figure 1:** Screening of 80 hybrid Citrus varieties and both parents (Fortune mandarin and Ellendale tangor) according to MDA content during warm period. The eight selected varieties are shown in red square. All data are mean values (± S.E.) of 4 independent biological replicates for each genotype (*n* = 4) obtained by pooling 15 fully-expanded leaves.

**Table 1.** Chemical composition of leaf essential oils of nine citrus varieties obtained from 3 independent biological replicates for each genotype (*n* = 3). Order of elution and percentages of individual components are given on non-polar (BP1) capillary column, those with an asterisk excepted, percentage on polar column (BP20). RI^a^, RI^p^: retention indices on non-polar and polar column, respectively. (CP, cold period; WP, warm period)

|  | **Retention indices (RI)** | | **Fortune mandarin** | | **Ellendale tangor** | | **D1-2x** | | **D39-2x** | | **T1-3x** | | **T2-3x** | | **T38-3x** | | **T40-3x** | | **Clementine** | |
| --- | --- | --- | --- | --- | --- | --- | --- | --- | --- | --- | --- | --- | --- | --- | --- | --- | --- | --- | --- | --- |
| **Compounds** | **RI^a^** | **RI^p^** | ***CP*** | ***WP*** | ***CP*** | ***WP*** | ***CP*** | ***WP*** | ***CP*** | ***WP*** | ***CP*** | ***WP*** | ***CP*** | ***WP*** | ***CP*** | ***WP*** | ***CP*** | ***WP*** | ***CP*** | ***WP*** |
| α-Thujene | 922 | 1016 | 0.47 | 0.28 | 0.52 | 0.3 | 0.4 | 0.3 | 0.48 | 0.24 | 0.4 | 0.24 | 0.27 | 0.18 | 0.58 | 0.04 | - | - | 0.42 | 0.28 |
| α-Pinene | 927 | 1014 | 1.9 | 1.34 | 1.92 | 1.45 | 1.67 | 1.41 | 1.86 | 1.24 | 1.35 | 1.08 | 0.98 | 0.81 | 1.91 | 0.32 | - | - | 1.6 | 1.2 |
| Camphene | 942 | 1061 | 0.05 | 0.04 | 0.05 | 0.04 | 0.05 | 0.04 | 0.06 | 0.04 | 0.04 | 0.04 | 0.03 | 0.03 | 0.05 | - | - | - | 0.04 | 0.04 |
| Sabinene | 966 | 1121 | 46.78 | 44.81 | 41.29 | 40.74 | 39.78 | 40.29 | 60.44 | 52.72 | 37.82 | 40.39 | 28.34 | 26.65 | 45.62 | 25.72 | 2.53 | 0.01 | 36.38 | 35.11 |
| β-Pinene | 969 | 1109 | 2.45 | 2.05 | 2.39 | 1.97 | 2.14 | 1.98 | 2.87 | 2.32 | 1.91 | 1.81 | 1.45 | 1.28 | 2.53 | 1.06 | 0.1 | - | 1.99 | 1.67 |
| Myrcene | 979 | 1157 | 3.26 | 2.72 | 3.74 | 3.16 | 2.82 | 2.54 | 3.65 | 3.61 | 2.85 | 2.91 | 1.91 | 1.78 | 3.31 | 1.88 | 0.67 | 0.33 | 3.2 | 2.9 |
| α-Phellandrene | 996 | 1162 | 0.06 | 0.07 | 0.38 | 0.38 | 0.09 | 0.07 | 0.08 | 0.1 | 0.23 | 0.24 | 0.07 | 0.05 | 0.11 | - | - | - | 0.43 | 0.53 |
| δ-3-Carene | 1004 | 1146 | 0.01 | 0.22 | 3.96 | 3.85 | 0.01 | 0.16 | - | 0.37 | 2.42 | 2.13 | - | - | 0.02 | - | - | - | 3.65 | 4.99 |
| α-Terpinene | 1008 | 1177 | 1.37 | 1.08 | 2.06 | 1.25 | 1.4 | 1.31 | 1.86 | 1.3 | 1.16 | 1.07 | 0.98 | 0.81 | 1.64 | 0.38 | 0.09 | - | 1.7 | 1.13 |
| p-Cymene | 1010 | 1268 | 0.37 | 0.04 | 0.3 | 0.05 | 0.12 | 0.02 | 0.28 | 0.06 | 0.37 | 0.06 | 0.23 | - | 0.62 | 0.15 | - | - | 0.02 | 0.06 |
| Limonene* | 1020 | 1198 | 1.84 | 1.49 | 0.07 | 4.15 | 1.52 | 1.25 | 1.82 | 2.42 | 8.6 | 4.55 | 1.4 | 1.05 | 1.79 | 1.23 | 2.11 | 0.94 | 3.04 | 3.03 |
| β-Phellandrene* | 1020 | 1208 | 0.59 | 0.51 | 5.35 | 0.64 | 0.5 | 0.49 | 0.6 | 0.64 | 0.65 | 0.67 | 0.36 | 0.32 | 0.58 | 0.39 | 0.02 | - | 0.82 | 0.79 |
| (Z)-β-Ocimene | 1024 | 1229 | 0.31 | 0.24 | 0.17 | 0.14 | 0.28 | 0.21 | 0.25 | 0.31 | 0.25 | 0.28 | 0.32 | 0.31 | 0.27 | 0.22 | 0.98 | 0.78 | 0.18 | 0.17 |
| (E)-β-Ocimene | 1035 | 1247 | 8.73 | 7.11 | 5.99 | 4.84 | 8.64 | 6.76 | 6.47 | 8.52 | 6.14 | 8.13 | 10.78 | 10.53 | 8.73 | 7.78 | 34.08 | 21.94 | 5.32 | 4.89 |
| γ-Terpinene | 1047 | 1242 | 2.54 | 1.86 | 3.45 | 2.02 | 2.32 | 2.17 | 3.19 | 2.6 | 2.08 | 1.95 | 1.78 | 1.38 | 3.17 | 1.33 | 0.41 | - | 2.79 | 1.86 |
| trans-Sabinene hydrate | 1052 | 1460 | 0.7 | 0.95 | 0.86 | 1.18 | 1.1 | 1.32 | 0.68 | 1.11 | 0.98 | 1.03 | 1 | 0.99 | 0.88 | 1.35 | 0.26 | - | 1.25 | 1.29 |
| Terpinolene | 1077 | 1279 | 0.61 | 0.5 | 1.43 | 1.18 | 0.55 | 0.55 | 0.76 | 0.76 | 0.84 | 0.86 | 0.44 | 0.33 | 0.76 | 0.35 | - | - | 1.24 | 1.27 |
| Linalool | 1083 | 1544 | 15.93 | 25.41 | 7.11 | 14.85 | 26.42 | 30.31 | 3.73 | 9.57 | 16.16 | 22.03 | 40.57 | 46.22 | 14.95 | 34.74 | 44.74 | 64.69 | 20.13 | 22.6 |
| cis-p-Menth-2-en-1-ol | 1106 | 1558 | 0.35 | 0.29 | 0.44 | 0.29 | 0.34 | 0.33 | 0.44 | 0.39 | 0.34 | 0.28 | 0.31 | 0.21 | 0.42 | 0.43 | 0.1 | - | 0.34 | 0.32 |
| trans-Menth-2-en-1-ol | 1121 | 1623 | 0.2 | 0.15 | 0.28 | 0.15 | 0.2 | 0.18 | 0.26 | 0.19 | 0.2 | 0.14 | 0.18 | 0.11 | 0.25 | 0.2 | - | - | 0.19 | 0.16 |
| Citronellal | 1129 | 1476 | 0.02 | 0.06 | 1.28 | 1.35 | 0.03 | 0.07 | 0.01 | 0.15 | 2.08 | 1.42 | 0.03 | 0.01 | 0.04 | - | - | - | 1.08 | 3.48 |
| Terpinen-4-ol | 1160 | 1597 | 5.54 | 4.41 | 7.09 | 4.48 | 5.29 | 5.17 | 7.36 | 5.9 | 5.31 | 4.17 | 4.87 | 3.22 | 6.47 | 6.45 | 2.14 | - | 5.27 | 4.64 |
| α-Terpineol | 1170 | 1691 | 0.3 | 0.46 | 0.36 | 0.93 | 0.62 | 0.96 | 0.3 | 0.43 | 0.52 | 0.51 | 0.59 | 0.67 | 0.38 | 0.82 | - | - | 0.77 | 0.99 |
| Myrtenol | 1178 | 1740 | 0.08 | 0.08 | 0.11 | 0.08 | 0.07 | 0.08 | 0.1 | 0.11 | 0.07 | 0.07 | 0.06 | 0.04 | 0.08 | 0.07 | - | - | 0.06 | 0.07 |
| Cis-piperitol | 1188 | 1673 | 0.11 | 0.05 | 0.15 | 0.06 | 0.1 | 0.06 | 0.15 | 0.08 | 0.11 | 0.05 | 0.1 | - | 0.14 | - | - | - | 0.1 | 0.05 |
| Citronellol | 1207 | 1760 | 0.01 | - | 0.51 | 0.39 | 0.01 | - | 0.01 | 0.04 | 0.52 | 0.24 | 0.02 | - | - | - | - | - | 0.26 | 0.6 |
| Neral | 1212 | 1678 | 0.03 | 0.05 | 0.19 | 1.22 | - | - | - | - | 0.41 | 0.09 | - | - | - | - | - | - | 0.02 | 0.23 |
| Geraniol | 1232 | 1842 | - | 0.02 | 0.08 | 0.39 | - | - | 0.03 | - | 0.11 | 0.02 | - | - | - | - | - | - | 0.01 | 0.06 |
| Geranial | 1240 | 1728 | 0.01 | 0.07 | 0.26 | 1.64 | - | - | 0.02 | - | 0.52 | 0.13 | - | - | 0.02 | - | - | - | - | 0.31 |
| 2-Methoxy-4-vinylphenol | 1280 | 2191 | 0.02 | - | 0.09 | 0.09 | 0.08 | 0.06 | 0.02 | - | - | 0.04 | - | 0.06 | - | - | - | - | 0.34 | 0.07 |
| Neryl acetate | 1340 | 1745 | - | - | 0.14 | 0.1 | - | - | - | - | 0.09 | 0.02 | - | - | - | 0.15 | - | - | - | - |
| Geranyl acetate | 1358 | 1752 | - | - | 0.21 | 0.46 | - | - | - | - | 0.35 | 0.11 | - | - | - | 0.26 | - | - | 0.05 | 0.39 |
| β-Elemene | 1384 | 1583 | 0.92 | 0.49 | 0.74 | 0.62 | 0.47 | 0.26 | 0.21 | 0.58 | 0.14 | 0.28 | 0.26 | 0.47 | 0.85 | 3.66 | 2.12 | 2.05 | - | 0.07 |
| (E)-β-Caryophyllene | 1412 | 1589 | 0.22 | 0.13 | 0.23 | 0.19 | 0.15 | 0.11 | 0.06 | 0.22 | 0.11 | 0.15 | 0.05 | 0.09 | 0.17 | 0.72 | 0.35 | 0.42 | 0.24 | 0.08 |
| (E)-β-Farnesene | 1445 | 1661 | 0.12 | 0.06 | 0.07 | 0.13 | 0.06 | 0.03 | 0.02 | 0.08 | 0.03 | 0.04 | 0.03 | 0.05 | 0.1 | 0.41 | 0.19 | 0.33 | 0.07 | 0.01 |
| (E,E)-α-Farnesene | 1492 | 1742 | 0.05 | 0.08 | 0.03 | 0.04 | 0.02 | - | - | - | 0.02 | 0.02 | - | - | 0.04 | 0.07 | - | 0.04 | - | - |
| β-Elemol | 1529 | 2072 | 0.04 | 0.04 | 0.05 | 0.05 | 0.03 | - | 0.02 | 0.05 | - | - | 0.04 | 0.05 | 0.04 | 0.15 | 0.11 | 0.16 | 0.02 | 0.01 |
| (E)-Nerolidol | 1543 | 2035 | 0.13 | 0.08 | 0.1 | 0.07 | 0.07 | 0.05 | 0.02 | 0.05 | 0.13 | 0.13 | 0.04 | 0.03 | 0.08 | 0.2 | 0.14 | 0.12 | 0.1 | 0.1 |
| Caryophyllene oxide | 1564 | 1974 | 0.05 | 0.02 | 0.02 | - | 0.03 | - | - | - | 0.03 | - | 0.02 | - | 0.02 | - | - | 0.08 | - | - |
| Selin-11-en-4-α-ol | 1631 | 2242 | 0.09 | - | 0.07 | - | 0.03 | - | - | 0.03 | - | - | 0.02 | 0.02 | 0.06 | 0.17 | 0.02 | 0.2 | - | - |
| β-Sinensal | 1669 | 2222 | 0.01 | 0.15 | 2.79 | 2.11 | 0.03 | 0.15 | - | 0.08 | 0.01 | 0.11 | 0.48 | 0.52 | 0.06 | - | 1.55 | 1.26 | 3.77 | 2.32 |
| α-Sinensal | 1721 | 2322 | 2.77 | 1.5 | 1.71 | 0.95 | 1.45 | 0.86 | 1.51 | 2.84 | 1.55 | 1.89 | 1.38 | 1.36 | 2.29 | 6.41 | 6.69 | 3.22 | 2 | 1.22 |
| (E)-Phytol | 2093 | 2605 | 0.22 | 0.15 | 0.16 | 0.31 | 0.13 | 0.16 | 0.14 | - | 0.09 | 0.15 | 0.13 | 0.21 | 0.22 | 2.52 | 0.53 | 1.1 | 0.17 | 0.24 |
| **Total** |  |  | 99.26 | 99.06 | 98.2 | 98.29 | 99.02 | 99.71 | 99.76 | 99.15 | 96.99 | 99.53 | 99.52 | 99.84 | 99.25 | 99.63 | 99.93 | 97.67 | 99.06 | 99.23 |


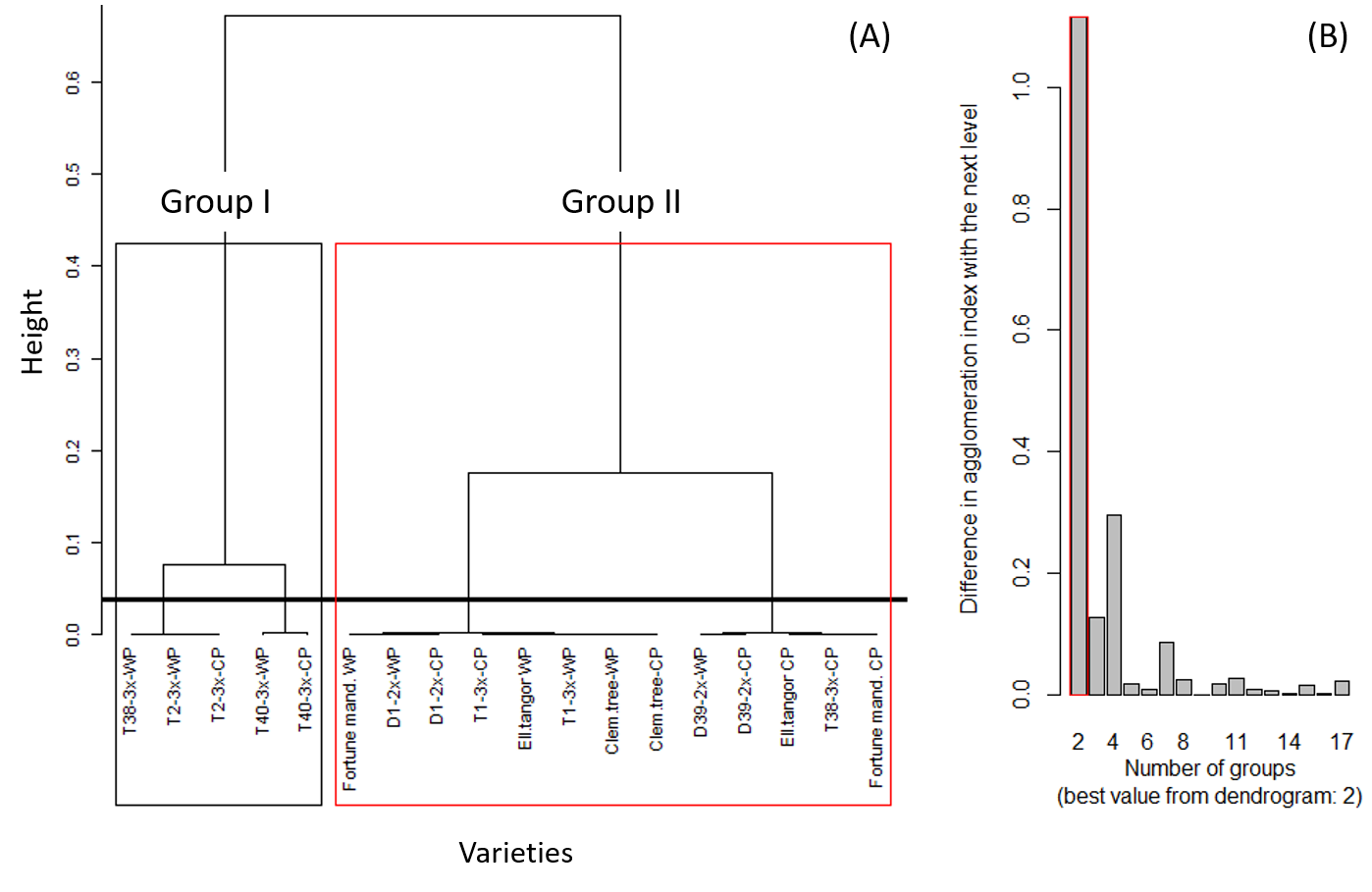


**Figure 2:** Cluster analysis following Ward’s method (euclidean distance) of the leaf essential oils based on the chemical composition of nine citrus varieties during cold period (CP) and warm period (WP). (A) Repartition to cluster dendrogramm and (B) best number of groups for the repartition. Dashed lines represent the division of the principal groups, each in two sub-groups resulting in four clusters corresponding to the second best group numbers.
